# The Reciprocal Relationship Between Parental eHealth Literacy Mediation and Adolescents’ eHealth Literacy: Three-Wave Longitudinal Study

**DOI:** 10.2196/67034

**Published:** 2025-06-09

**Authors:** Natalie Tercova, Michal Muzik, Lenka Dedkova, David Smahel

**Affiliations:** 1 Interdisciplinary Research Team on Internet and Society Faculty of Social Studies Masaryk University Brno Czech Republic

**Keywords:** eHealth literacy, parental mediation, longitudinal research, adolescents, eHealth mediation, health information

## Abstract

**Background:**

The online environment provides adolescents with vast amounts of health-related information; however, navigating this effectively requires high levels of eHealth literacy to avoid misinformation and harmful content. Parental guidance is often considered a crucial factor in shaping adolescents’ online health behaviors; however, there is limited longitudinal research examining how parental eHealth literacy mediation influences adolescents’ development of eHealth literacy over time.

**Objective:**

This study aims to examine the reciprocal relationship between parental eHealth literacy mediation and adolescents’ eHealth literacy. It also investigates whether parental education moderates this relationship, specifically exploring whether higher levels of parental education enhance the effectiveness of eHealth literacy mediation in improving adolescents’ eHealth literacy.

**Methods:**

A 3-wave longitudinal study was conducted, collecting data from 2500 adolescent-parent pairs. A random intercept cross-lagged panel model was applied to assess the reciprocal effects between parental eHealth literacy mediation and adolescents’ eHealth literacy across the 3 waves. Parental education was included in the model as a potential moderating variable to examine whether it influences the strength of the relationship between parental eHealth literacy mediation and adolescents’ eHealth literacy.

**Results:**

The findings revealed no significant within-person effects, indicating that changes in parental eHealth literacy mediation over time did not lead to corresponding changes in adolescents’ eHealth literacy (T1→T2 β=–.03, *P*=.65; T2→T3 β=.01, *P*=.84), and vice versa (T1→T2 β=.02, *P*=.71; T2→T3 β=–.07, *P*=.19). Furthermore, the data did not support a moderating effect of parental education, suggesting that higher educational attainment does not enhance the impact of parental eHealth literacy mediation. However, a significant between-person association was observed: adolescents with higher levels of eHealth literacy tend to have parents who engage more frequently in eHealth literacy mediation (r=0.30, *P*<.001).

**Conclusions:**

This study contributes to the understanding of parental involvement in shaping adolescents’ eHealth literacy. Contrary to expectations, parental eHealth literacy mediation does not appear to have a significant longitudinal impact on the development of adolescents’ eHealth literacy, nor does higher parental education strengthen this relationship. These findings suggest that additional factors beyond parental mediation and education may play a critical role in supporting adolescents’ ability to navigate online health information effectively.

## Introduction

### Adolescents, Digital Health Information, and Parental Mediation

The online environment provides adolescents with unprecedented access to a wide range of information, including content related to health [1]. As frequent users of digital media, adolescents increasingly turn to online sources to inform their health-related decisions [2]. While this access can be empowering, it also carries risks. For instance, adolescents may encounter health information that is false, misleading, or even harmful, with potentially serious consequences [3]. Much of this content is now encountered passively through algorithm-curated feeds on platforms such as TikTok (ByteDance Ltd), Instagram (Meta Platforms, Inc), or YouTube (Alphabet Inc), which prioritize engagement over accuracy [4]. Influencer-driven health messaging—often lacking scientific grounding—can go viral and shape adolescent attitudes and behaviors in ways that are not always beneficial [5].

This risk was further amplified during the COVID-19 pandemic, which intensified young people’s reliance on digital media for health-related information while simultaneously exposing them to a surge of misinformation [6]. Studies show that adolescents with lower eHealth literacy were more vulnerable to misinformation and more likely to adopt maladaptive health behaviors during this time [7-9]. Recent data from the World Health Organization [10] indicate that many European adolescents face difficulties both in finding online health information and in evaluating its reliability, underscoring persistent gaps in essential eHealth literacy skills. These developments highlight the urgent need to equip adolescents with the skills to navigate digital health information critically and responsibly.

In this context, eHealth literacy—defined as the knowledge and skills needed to locate, understand, evaluate, and apply online health information to support health and prevent illness [11,12]—becomes a critical competency. Prior research has shown that individuals with higher levels of eHealth literacy are more likely to actively seek health information [13], engage in healthier lifestyle behaviors [14], and attend preventive medical appointments, such as screenings [15]. Beyond individual health outcomes, adolescent eHealth literacy also contributes to broader public health goals by enhancing collective resilience against misinformation and supporting informed community health decisions [6,16]. Given these benefits, fostering eHealth literacy in adolescence is essential for supporting informed and health-conscious decision-making from an early age.

Parental mediation—defined as the parenting practice that aims to enhance children’s understanding of media content [17]—is one way to increase eHealth literacy among adolescents. However, our understanding of the contributions parents make to the development of eHealth literacy in adolescents remains limited. Research indicates that parental involvement and guidance significantly shape children’s media use and health-related behaviors [18,19]. Nevertheless, there is a shortage of longitudinal studies that verify the actual impact of parental mediation.

At the same time, it is recognized that parents’ literacy and education—particularly in the field of health—are associated with their ability to convey accurate information [20]. More educated parents may therefore be more effective in helping their adolescent children acquire relevant skills. This may be due not only to their stronger health knowledge, but also to greater digital confidence [21], a tendency to model critical thinking in everyday interactions [22], and more frequent engagement in reflective conversations about online content [23]. These factors suggest that parental education may facilitate more active and effective mediation of adolescents’ encounters with digital health information. However, empirical evidence remains limited, and it is unclear whether this principle extends to parental eHealth literacy mediation (hereafter referred to as eHealth literacy mediation). Moreover, adolescents’ eHealth literacy may, in turn, influence eHealth literacy mediation. When deciding on mediation strategies, parents may consider their child’s level of literacy and adjust their efforts accordingly. However, most current research is cross-sectional, capturing primarily between-person effects, and there is a lack of longitudinal research examining within-person effects in eHealth literacy mediation. The expectation of reciprocal effects is further supported by the Self-Determination Theory [24], which emphasizes the dynamic nature of parent-child interactions and suggests that these relationships involve bidirectional influence. Adolescents’ media-related behaviors—such as their choices regarding online health information—may shape how parents engage in eHealth literacy mediation.

Our study focuses on the reciprocal dynamics between eHealth literacy mediation and adolescents’ eHealth literacy, while also considering the moderating role of parental education. We use dyadic data from Czech parents and their adolescent children, collecting parent-reported data on eHealth literacy mediation and adolescent-reported data on eHealth literacy. This design allows us to examine whether changes in parental mediation practices are associated with subsequent changes in adolescents’ eHealth literacy, and conversely, whether adolescents’ literacy levels influence the extent to which parents engage in mediation. By adopting a longitudinal perspective, the study aims to clarify how these processes unfold over time within families and whether their strength varies according to parental educational background.

### Theoretical Background

#### Adolescents and eHealth Literacy

Adolescents significantly incorporate digital technologies into their daily lives—they are frequent internet users and engage in a wide range of online activities [25]. This includes activities related to health. Existing studies show that adolescents regularly search for various types of health information online [26-28], including sexual and reproductive health topics [29], as well as information about diseases and fitness [30]. Adolescents report that they find online health information useful [31]. However, the quality of this information varies, and they may encounter inaccurate or false content [32].

Adolescents may therefore face challenges in navigating online health information effectively, often due to gaps in their eHealth literacy [27]. Successfully navigating the complex digital environment requires more than just basic skills—it demands digital literacy, which includes not only functional abilities but also a critical understanding of the digital landscape [33]. eHealth literacy, a specific dimension of digital literacy, refers to the ability to access, understand, evaluate, and apply online health information to promote personal health and prevent illness [12,34,35]. Higher levels of eHealth literacy have been associated with more frequent access to reliable health information [13], greater confidence in making informed health decisions [15], and improved overall health outcomes [36]. Systematic reviews have shown that health literacy is associated with better understanding of health information [37], greater treatment adherence [38], improved quality of life [39], and several other health-related outcomes among adults. Similar findings apply to eHealth literacy. A recent meta-analysis [40] focusing on individuals under the age of 65 years reported a pooled correlation of 0.28 between eHealth literacy and health-related behaviors, with significant positive associations found in 22 of the 29 included studies. Among adolescents specifically, a systematic review [41] found that higher levels of health literacy were positively associated with health-promoting behaviors (eg, preventive behaviors, nutrition, exercise) and negatively associated with substance use (eg, smoking, binge drinking). While general health literacy and eHealth literacy are conceptually distinct constructs, findings from the broader health literacy literature may illuminate important patterns relevant to eHealth literacy.

Empirical evidence on eHealth literacy among adolescents specifically is more limited. Kim et al [40] identified a significant correlation between eHealth literacy and health-related behaviors, although this relationship was weaker in younger populations. Despite often reporting high confidence in their ability to search for online health information, studies by Fleary et al [41] and Sansom-Daly et al [42] suggest that adolescents frequently struggle to critically evaluate the information they encounter. Similarly, Taba et al [9] found that while adolescents feel confident using online health information, they often overestimate their actual eHealth literacy.

Gulec et al [28] found that adolescents’ eHealth literacy is closely associated with their online health information–seeking behaviors, particularly regarding disease and fitness. Research by Tümer and Sümen [43] suggested that adolescents from nuclear families with easy internet access; an understanding of the importance of good health; and well-educated, financially stable parents tend to have higher levels of eHealth literacy. Additionally, Norman and Skinner [34] observed that females generally exhibit slightly higher eHealth literacy scores than males, although this finding is not specific to adolescents.

Furthermore, studies by Cheng et al [44] and Korkmaz Aslan et al [45] emphasized the need for targeted educational interventions to enhance adolescents’ critical appraisal skills, enabling them to navigate and apply online health information more effectively. Regarding broader digital literacy, adolescents’ digital skills have been found to correlate positively with their frequency of searching for health information online [46].

As systematic evidence shows that health literacy is positively associated with health-related behaviors [40,41], attention should be directed toward factors that may enhance eHealth literacy among adolescents. Various mechanisms may promote adolescents’ eHealth literacy, including the potential role of parental mediation.

#### The Role of Parental Mediation

Parents are primary socializing agents who influence their children’s behavior, attitudes, and values through their parenting practices [18,47]. This influence extends to media use, as adolescents often mirror their parents’ media consumption patterns [48]. Consequently, the extent to which children adopt attitudes and behaviors presented in the media depends in part on their parents’ activities, which shape how children access, receive, process, and respond to information [49]. A specific form of media parenting is parental mediation, defined as the information and communication technology (ICT)–specific parenting practices that parents use to maximize the benefits of, and minimize the risks associated with, their children’s online experiences [17,50].

Several types of parental mediation have been commonly researched, although no single classification or measurement approach is universally accepted [51,52]. Parental mediation can be conceptualized and measured broadly, as a general parental approach to all ICT-related issues, or more specifically as active or restrictive mediation—2 of the most frequently studied strategies [53]. Active mediation involves parents engaging in conversations with their children about online activities, discussing and sharing those experiences, providing guidance on internet use, and explaining how to navigate challenging situations [54]. By contrast, restrictive mediation involves setting rules for a child’s media use by limiting the time, place, or circumstances of media consumption without necessarily providing explanations for these restrictions [55]. Restrictive parental mediation has been associated with lower digital skills in children [56,57]. Conversely, active parental mediation has demonstrated a positive effect on children’s online activities and digital skills [58,59]. However, these associations tend to be weak, highlighting the need for longitudinal research to better understand the impact of active parental mediation on digital skills [60].

Another way to conceptualize parental mediation is by focusing on the specific activity it targets rather than the ICT domain as a whole, such as mediation of video gaming [61], social networking [62], or online interactions [63]. The advantage of this approach is that the relationship between the specific type of mediation and the related children’s outcomes is more clearly theoretically grounded, making it easier to apply the findings to practical contexts. In our study, we adopt this activity-specific approach by focusing specifically on eHealth literacy mediation, an active mediation strategy aimed at improving children’s understanding, seeking, and critical evaluation of online health information. Unlike general or risk-focused mediation, which often centers on screen time limits or content restrictions, eHealth literacy mediation is a skill-based, educational approach designed to strengthen adolescents’ ability to navigate complex digital health environments. It involves discussions not only about safety but also about credibility, evidence, and the trustworthiness of sources. Limited research has examined this specific type of parental mediation. Previous studies found a positive association between eHealth literacy mediation and the frequency of adolescents’ online health information–seeking behaviors [19]. Additionally, active parental mediation—assessed as a general practice—was positively linked to adolescents’ eHealth literacy [64]. However, these are cross-sectional studies. Beyond these studies, research shows that adolescents trust their parents as their primary source of health information [65] and rely on parental guidance to critically evaluate the quality of health information they encounter [66]. Additionally, Gulec et al [28] provided evidence highlighting the significant role of parental online health information–seeking mediation in increasing adolescents’ online health information–seeking behaviors. As mediation aims to influence children’s behavior and literacy, we expect that eHealth literacy mediation can positively impact adolescents’ eHealth literacy.

#### The Reciprocal Relationship Between eHealth Literacy Mediation and eHealth Literacy in Adolescents

eHealth literacy mediation may not only impact the eHealth literacy of parents’ children but may also be influenced by the children’s own eHealth literacy. We can assume that adolescents’ eHealth literacy affects parents’ decisions to engage in eHealth literacy mediation. Qualitative studies highlight that parental mediation arises from everyday interactions between parents and their children [67] and should be understood as a dynamic, cocreated process involving both parties [68,69]. Quantitative studies examining predictors of parental mediation report similar findings: the level of parental mediation depends, among other factors, on children’s characteristics and usage patterns. For example, parents tend to reduce mediation as children grow older, particularly during adolescence [70,71], and adjust their mediation based on children’s frequency of online activities [72] or their own assessment of whether the internet is having a negative impact on their child and how well the child can self-regulate [73].

Similarly, parents’ perceptions of adolescents’ digital literacy may influence their engagement in eHealth literacy mediation. Previous research [50] has shown that children’s digital skills predict parental mediation. Thus, when parents observe their adolescent struggling to understand or search for quality health information online (ie, demonstrating low eHealth literacy), they may increase their mediation efforts. Conversely, if an adolescent exhibits higher eHealth literacy, parents may feel that mediation is less necessary in this area and subsequently reduce their involvement.

Consequently, we anticipate a reciprocal relationship between these 2 variables over time. Our objective is to investigate the potential longitudinal association between eHealth literacy mediation and adolescents’ eHealth literacy. Therefore, we examine the following hypotheses:

- H1: A change in eHealth literacy mediation will be followed by a change in the adolescent’s eHealth literacy 6 months later.
- H2: A change in the adolescent’s eHealth literacy will be followed by a change in eHealth literacy mediation 6 months later.

#### Parental Educational Level and eHealth Literacy

Studies assessing the impact of parental mediation on children’s outcomes often report inconsistent empirical evidence regarding its effectiveness (see reviews [53,74]). One possible reason is that the same parenting practices may not be equally effective for every child (ie, their effects may be conditional). For example, a 2-wave study found that parental restrictions on video gaming effectively reduced children’s aggressive behavior only in families with authoritarian or authoritative parenting styles, but not in those with permissive or neglectful styles [75]. On a similar note, Valkenburg et al [76] argued that the effectiveness of parental mediation depends on how it is delivered. They distinguished among autonomy-supportive, controlling, and inconsistent styles of both restrictive and active parental mediation. Thus, the manner in which parents deliver mediation may influence how their efforts are received and, consequently, how these efforts are reflected in children’s outcomes.

Given that recognizing the quality of online health information can be considered an advanced digital skill, requiring relatively specific knowledge and competencies, we focus on parental education as a potential moderator. Previous studies have shown that individuals with limited education [77,78] are more likely to have inadequate eHealth literacy skills. Parents with lower education and literacy levels tend to have less health knowledge and engage in behaviors that are less beneficial for their children’s health compared with parents with higher literacy levels [79]. Similarly, children of parents with lower literacy often experience poorer health outcomes [79]. Therefore, a parent’s literacy and knowledge, especially in the health domain, may significantly influence their ability to convey accurate information that children can effectively use [20].

Furthermore, studies have shown that more educated parents tend to be more confident in their digital skills and are more capable of effectively prioritizing active mediation on various digital topics [21,50]. By contrast, less educated parents often perceive a generational gap in which their children are seen as more digitally knowledgeable or competent [21]. As a result, more educated parents may be better equipped to deliver eHealth literacy mediation with greater knowledge, accuracy, and confidence. They may also place greater value on critical thinking and the evaluation of health-related sources than less educated parents [80]. This awareness can shape their approach to eHealth literacy mediation strategies. Conversely, less educated parents may be perceived by their children as less proficient in this area, potentially undermining the authority and credibility of their guidance.

Therefore, parents’ education levels may moderate the relationship between eHealth literacy mediation and their children’s eHealth literacy. This could either amplify or attenuate the effectiveness of parental mediation strategies in shaping adolescents’ eHealth literacy development. Accordingly, we formulate the following hypothesis:

- H3: The effect of eHealth literacy mediation by a parent with a higher level of education on the adolescent’s eHealth literacy will be greater than that of a parent with a lower level of education.

Given the variability in findings across studies reviewed above, these inconsistencies may partly stem from differences in how eHealth literacy and related outcomes are measured. Variations in instruments or self-report formats can influence both the strength and direction of observed associations, making cross-study comparisons inherently difficult.

### Confounding Variables

#### Adolescent’s Age

Previous research focusing on adolescents’ digital literacy has provided strong evidence that digital skills tend to improve with age [81-83]. However, findings regarding health literacy are more mixed: while some studies report a positive relationship with age, others do not find a significant association [41]. Kim et al [40], for instance, observed that younger populations exhibit a weaker correlation between eHealth literacy and health-related behaviors. Given that our sample includes individuals in early to middle adolescence—a developmental period marked by significant cognitive and social maturation—we expect that eHealth literacy will increase with age in our study. Furthermore, previous research has shown that parents tend to reduce their mediation efforts as children grow older [70,71]. In light of this, we will consider adolescents’ age as a potential confounding variable in our analyses.

#### Adolescent’s Gender

Previous research on digital literacy among adolescents has highlighted the potential influence of gender. Several studies have found that girls tend to demonstrate higher digital skills than boys [84,85]. Conversely, other studies report the opposite trend, indicating that boys may exhibit higher digital skills than girls [86,87]. Similarly, findings on the relationship between gender and health literacy remain inconsistent. Of the 13 studies reviewed by Fleary et al [41], 9 found no significant relationship between gender and health literacy [64]. However, 3 studies reported higher health literacy among girls [88-90], while 1 study found higher health literacy among boys [91]. Regarding eHealth literacy specifically, Norman and Skinner [34] suggested that females tend to score slightly higher than males. Furthermore, previous research has indicated that parents tend to engage in more mediation with girls than with boys [50]. Therefore, in this study, we include the adolescent’s gender as a confounding variable.

### Study Overview

In this study, we examine the reciprocal dynamics between eHealth literacy mediation (as reported by parents) and adolescents’ eHealth literacy (as reported by the adolescents themselves) using a longitudinal research design. We consider the moderating role of parental education and apply a within-person approach, which enables the analysis of individual-level changes and variations in both eHealth literacy mediation and adolescents’ eHealth literacy over time within the same participants. Specifically, we examine whether increases in eHealth literacy mediation lead to subsequent increases in adolescents’ eHealth literacy, and conversely, whether increases in adolescents’ eHealth literacy influence the extent of parental eHealth literacy mediation. By focusing on within-person dynamics, we aim to better disentangle the complexities of these associations and clarify the potential causal mechanisms underlying changes in adolescents’ eHealth literacy. Additionally, we examine the moderating role of parental education to test whether higher education strengthens the effectiveness of eHealth literacy mediation. The model also controls for adolescents’ age and gender.

## Methods

### Sample

The data for this study came from a survey evaluating various aspects of adolescents’ use of ICTs and their impact on well-being. Because of the COVID-19 pandemic, we selected an online panel for participant recruitment and used computer-assisted web interviewing for data collection. An external research agency in the Czech Republic managed the sampling and data collection. Eligible participants were Czech households with at least one adolescent aged 11-16 years and a caregiver, enabling data collection from adolescent-parent dyads within the same household.

We utilized quotas based on region (Nomenclature of Territorial Units for Statistics level 3 [NUTS3]), municipality size, and household socioeconomic status (proxied by parents’ education) to ensure proportional representation of Czech households with children. Additionally, quotas for adolescents’ age and gender were established to achieve a balanced distribution. Data were collected across 3 waves, spaced 6 months apart, beginning in June 2021 (time 1 [T1]) and concluding in June 2022 (T3).

The full sample for this study comprised 2500 adolescent-parent dyads at T1. At T1, of the 2500 adolescents, 1250 (50%) were girls (mean_age_ 13.4 years, SD 1.7 years), and 1592 out of 2500 (63.68%) parents were women (mean_age_ 42.7 years, SD 7.1 years). At T2, the sample included 1654 dyads (adolescents: girls, 800/1654, 48.37%, mean_age_ 13.4 years; parents: women, 1004/1654, 60.70%, mean_age_ 43.3 years), and at T3, 1102 dyads (adolescents: girls, 532/1102, 48.28%, mean_age_ 13.4 years; parents: women, 661/1102, 59.98%, mean_age_ 43.3 years). Retention rates were over 66% from T1 to T2 (1654/2500, 66.16%) and from T2 to T3 (1102/1654, 66.63%). See the “Analysis” section for details on attrition.

### Ethics Approval

The study protocol was approved by the Research Ethics Committee at Masaryk University (EKV-2018-068). Before participation, respondents were informed about the nature and purpose of the survey, their right to decline involvement, and their ability to skip any questions by selecting the “I prefer not to say” option available for all items. Informed consent was obtained from both parents and adolescents. Participants were rewarded with reward points redeemable for approximately US $4.

### Measures

*eHealth literacy mediation* was measured with the following: “Children can find/encounter various health-related information on the internet. In the past 6 months, how often have you talked to your child about...” followed by 4 statements (eg, How can we tell that health-related information on the internet is true or false?; How should we evaluate the quality of such health-related information on the internet?). These items were previously used in the study by Gulec et al [28] to capture parental advice regarding the critical evaluation and recognition of online health information. Parents responded using a 6-point scale ranging from 1 (never) to 6 (several times a day). The internal consistency was excellent, with ω=0.95 at T1, ω=0.96 at T2, and ω=0.96 at T3. Descriptive statistics for the scale are presented in Table 1. The results supported longitudinal metric invariance, as indicated by a nonsignificant difference between the configural and metric models (Δχ^2^_6_=2.812, *P*=.83).

**Table 1:** Descriptive statistics.

Measure	n	M/Mo^a^	SD
T1 eHealth literacy	2418	3.34	0.88
T1 eHealth literacy mediation	2390	2.34	1.04
T2 eHealth literacy	1626	3.38	0.85
T2 eHealth literacy mediation	1623	2.44	1.06
T3 eHealth literacy	1091	3.47	0.81
T3 eHealth literacy mediation	1080	2.43	1.04
Parental education	2500	2	N/A^b^

^a^The M/Mo column represents the mode for parental education and means for the rest of the variables.

^b^N/A: not applicable (as parental education represents a categorical variable, no SD was reported).

*Adolescents’ eHealth literacy* was measured with 5 items from the Norman and Skinner [34] eHealth literacy scale and the following question: “One can find various health-related information on the internet. How true are the following statements for you?” Adolescents reported how strongly they agreed or disagreed with 5 statements (eg, I know where to find helpful health resources on the internet; I have the skills I need to evaluate the health resources I find on the internet) on a scale that ranged from 1 (strongly disagree) to 5 (strongly agree). The internal consistency was ω=0.92 for T1, ω=0.93 for T2, and ω=0.93 for T3. The results supported the longitudinal metric invariance (difference between the configural and the metric model: Δχ^2^_6_=9.334, *P*=.16).

*Parent’s education* was assessed with the following question: “What is your highest level of completed education?” Parents were asked to respond on a scale that ranged from 1 (unfinished primary) to 5 (university, including higher vocational school). To examine the role of parental education, participants were categorized based on the parent’s education level reported at T1 into 3 groups: without a maturity diploma (735/2500, 29.40%), with a maturity diploma (1118/2500, 44.72%), and with a university degree (647/2500, 25.88%). Within the Czech educational system, obtaining a maturity diploma and pursuing university studies represent key milestones that signify different levels of educational attainment.

The age and gender of the adolescent were indicated at T1.

### Analysis

We analyzed the longitudinal data using structural equation modeling in lavaan 0.6-18 [92], applying the maximum likelihood estimator with robust standard errors. All data analyzed during this study are included in this published article and Multimedia Appendices 1 and 2. Structural equation modeling enables simultaneous estimation of multiple relationships between observed and latent variables while accounting for measurement error. To test our hypotheses, we used a random intercept cross-lagged panel model (RI-CLPM). RI-CLPM is particularly suitable for longitudinal research questions, as it decomposes observed variance into within-person (ie, autoregressive and lagged effects) and between-person levels (ie, random intercepts), enabling a clearer understanding of temporal dynamics and individual changes over time [93]. Because of this decomposition, autoregressive effects are usually weaker in RI-CLPM and, rather than indicating stability, reflect within-person carry-over effects.

The RI-CLPM was recursive and included 2500 observations and 38 free parameters. We also included the child’s age and gender at T1 as time-invariant covariates. All variables were specified as manifest. For the attrition analysis, we conducted a multivariate analysis of variance. Participants who completed all waves differed overall from dropouts in demographic variables and the variables of interest (Pillai V=0.09, *F*_8,4628_=2.51, *P*=.01). The results of a separate univariate ANOVA revealed a significant difference only in eHealth literacy mediation (*F*_2,2316_=4.56, *P*=.01). Further analysis showed that this difference was driven by participants who took part only in T1 and T2 (mean_T12_ 2.47), who had the highest mean score in eHealth literacy mediation compared with those who participated only in T1 (mean_T1_ 2.30, *P*=.04), and those who participated in all waves (mean_T123_ 2.33, *P*=.02). To address this, we used full information maximum likelihood to handle missing data.

To test H3, we used a multigroup version of the RI-CLPM described above. We tested for differences in path estimates across groups using a likelihood-ratio test, which compares the fit of nested models to determine whether constraining specific paths significantly worsens model fit.

## Results

Table 1 presents the descriptive statistics, and Table 2 displays the intercorrelations between the variables. All variables were positively correlated, with the strongest correlations observed between the same variables measured at different time points.

The fit indices of our model are satisfactory (*χ*^2^_9_=12.10, *P*=.21, Comparative Fit Index=0.99, Tucker-Lewis Index=0.99, root mean square error of approximation=0.02, 90% CI 0.00-0.04, standardized root mean square residual=0.01).

**Figure 1:**
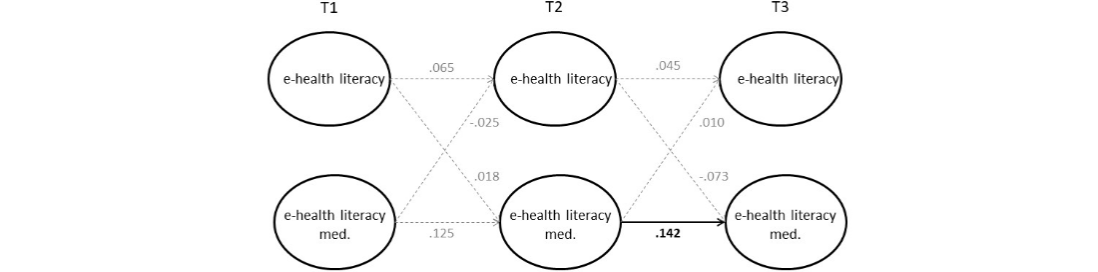
Within-person part of the RI-CLPM (random intercept cross-lagged panel model; N=2500). Med: mediation.

The model estimates are presented in Table 3, and the standardized lagged and autoregressive effects from the RI-CLPM are shown in Figure 1. The results indicate no significant causal effects, meaning that neither H1 (T1→T2 β=–.03, *P*=.65; T2→T3 β=.01, *P*=.84) nor H2 (T1→T2 β=.02, *P*=.71; T2→T3 β=–.07, *P*=.19) is supported. Among the control variables, only age showed significant positive effects on both eHealth literacy (β=.21, *P*<.001) and eHealth literacy mediation (β=.05, *P*=.01). In addition to these results, the correlation between the random intercepts of eHealth literacy and eHealth literacy mediation was significant (*r*=0.30, *P*<.001), indicating a meaningful between-person association—adolescents with higher eHealth literacy also tend to experience higher levels of eHealth literacy mediation.

**Table 2:** Correlations (Pearson r and 2-tailed *P* value).

Measure		T1 eHealth literacy	T1 eHealth literacy mediation	T2 eHealth literacy	T2 eHealth literacy mediation	T3 eHealth literacy	T3 eHealth literacy mediation	Parental education	
**T1 eHealth literacy**									
	*r*	1	0.189	0.454	0.159	0.436	0.110	0.002	
	*P* value	—^a^	<.001	<.001	<.001	<.001	<.001	.94	
**T1 eHealth literacy mediation**									
	*r*	0.189	1	0.143	0.602	0.139	0.528	–0.022	
	*P* value	<.001	—	<.001	<.001	<.001	<.001	.45	
**T2 eHealth literacy**									
	*r*	0.454	0.143	1	0.202	0.474	0.105	0.044	
	*P* value	<.001	<.001	—	<.001	<.001	<.001	.35	
**T2 eHealth literacy mediation**									
	*r*	0.159	0.602	0.202	1	0.151	0.583	–0.002	
	*P* value	<.001	<.001	<.001	—	<.001	<.001	.73	
**T3 eHealth literacy**									
	*r*	0.436	0.139	0.474	0.151	1	0.131	–0.004	
	*P* value	<.001	<.001	<.001	<.001	—	<.001	.72	
**T3 eHealth literacy mediation**									
	*r*	0.110	0.528	0.105	0.583	0.131	1	–0.041	
	*P* value	<.001	<.001	<.001	<.001	<.001	—	.04	
**Parental education**									
	*r*	0.002	–0.022	0.044	–0.002	–0.004	–0.041	1	
	*P* value	.93	.28	.08	.93	.89	.17	—	

^a^Not applicable.

**Table 3:** Estimates for RI-CLPM^a^.

Regression path		Estimate	SE	*P* value	*β*
**T2 eHealth literacy**					
	T1 eHealth literacy	0.062	.053	.24	.065
	T1 eHealth literacy mediation	–0.023	.051	.65	–.025
	Age	0.106	.009	<.001	.212
	Gender	–0.019	.029	.51	–.011
**T3 eHealth literacy**					
	T2 eHealth literacy	0.042	.062	.50	.045
	T2 eHealth literacy mediation	0.009	.044	.84	.010
	Age	0.106	.009	<.001	.222
	Gender	–0.019	.029	.51	–.012
**T2 eHealth literacy mediation**					
	T1 eHealth literacy	0.020	.052	.71	.018
	T1 eHealth literacy mediation	0.129	.081	.11	.125
	Age	0.028	.011	.01	.045
	Gender	–0.015	.038	.70	–.007
**T3 eHealth literacy mediation**					
	T2 eHealth literacy	–0.082	.063	.19	–.073
	T2 eHealth literacy mediation	0.142	.065	.03	.142
	Age	0.028	.011	.01	.044
	Gender	–0.015	.038	.70	–.007

^a^RI-CLPM: random intercept cross-lagged panel model.

The multigroup model analysis showed that no group exhibited a significant effect (without maturity diploma, T1 eHealth literacy mediation→T2 eHealth literacy β=–.05, *P*=.65, T2 eHealth literacy mediation→T3 eHealth literacy β=–.04, *P*=.71, T1 eHealth literacy→T2 eHealth literacy mediation β=–.07, *P*=.47, T2 eHealth literacy→T3 eHealth literacy mediation β=–.03, *P*=.84; with maturity diploma: T1 eHealth literacy mediation→T2 eHealth literacy β=–.00, *P*=.97, T2 eHealth literacy mediation→T3 eHealth literacy β=–.02, *P*=.85, T1 eHealth literacy→T2 eHealth literacy mediation β=–.04, *P*=.62, T2 eHealth literacy→T3 eHealth literacy mediation β=–.11, *P*=.19; with a university degree: T1 eHealth literacy mediation→T2 eHealth literacy β=–.03, *P*=.77, T2 eHealth literacy mediation→T3 eHealth literacy β=.04, *P*=.67, T1 eHealth literacy→T2 eHealth literacy mediation β=–.05, *P*=.53, T2 eHealth literacy→T3 eHealth literacy mediation β=–.07, *P*=.45), nor were there significant differences between groups (Δ*χ*^2^_8_=4.11, *P*=.85). Therefore, H3 was not supported by the results.

Regarding the stability of the variables, only the autoregressive effect of eHealth literacy mediation from T2 to T3 was significant (b=0.14, *P*=.03). This indicates that deviations in eHealth literacy mediation above or below an individual’s person-specific mean are associated with similar deviations in the same variable at a later time.

## Discussion

### Principal Findings

This study explored the reciprocal dynamics between eHealth literacy mediation and adolescents’ eHealth literacy, considering the moderating role of parental education. We found a significant between-person association—adolescents with higher eHealth literacy tended to have parents who engaged more frequently in eHealth literacy mediation—which aligns with findings from existing cross-sectional studies [50,64]. However, contrary to our expectations, we found no within-person associations, meaning that changes in eHealth literacy mediation were not followed by the anticipated increases in adolescents’ eHealth literacy, nor vice versa. This contrast suggests that although some families maintain consistently higher levels of both eHealth literacy mediation and adolescent eHealth literacy compared with others (between-person level), these differences appear relatively stable and are not easily influenced over time (within-person level). While we did not observe significant cross-lagged effects between eHealth literacy mediation and adolescents’ eHealth literacy, our results revealed a significant autoregressive effect for eHealth literacy mediation between T2 and T3. This suggests that short-term fluctuations in parental mediation tend to persist over time within individuals, indicating some intraindividual stability in how parents engage with their children around online health information. Taken together, these findings imply that simply increasing parental mediation efforts at a single point in time may not necessarily lead to improvements in adolescent eHealth literacy, particularly among older adolescents. From a policy and intervention perspective, this underscores the importance of sustained, developmentally appropriate strategies that extend beyond eHealth literacy mediation alone.

One possible reason for the lack of an apparent impact of parental efforts on adolescents’ skills is that, based on previous findings [53], adolescents in this age group are already quite autonomous and may not notice or acknowledge parental mediation efforts. This can be understood through the lens of cognitive development: as adolescents acquire more advanced reasoning skills, they tend to question authority and assert their independence [94]. Such autonomy needs may conflict with parental mediation efforts, especially when these efforts are perceived as controlling. This notion is further supported by studies comparing parents’ and adolescents’ perspectives, which reveal substantial discrepancies in their reports. A meta-analysis of parent-adolescent discordance in parenting variables found only a moderate correlation between their reports (pooled *r*=0.28) [95]. For instance, in a study on parental mediation of video games [96], parents reported a higher frequency for all 3 examined mediation strategies—active, restrictive, and coplaying. Similarly, Beyens and Valkenburg [97] found that parents reported greater use of autonomy-supportive strategies but lower use of controlling or inconsistent approaches. These findings suggest that the extent of parenting efforts, as perceived by parents, is not always recognized by their children, which may limit their effectiveness. Moreover, our study relies solely on parent-reported data. Prior research has shown that parents and adolescents often differ in their perceptions of mediation, with parents frequently reporting higher levels of engagement than their children perceive [97]. Future studies should therefore adopt a dual-informant approach to capture both perspectives and better reflect the mediation process as experienced by adolescents. Additionally, the relatively low levels of online parental mediation in the Czech Republic compared with the rest of Europe [25] could further reduce the potential impact of mediation in this context. Future research may thus consider whether similar patterns emerge in other cultural contexts with different digital parenting norms.

It is also possible that adolescents’ eHealth literacy is influenced more by socialization agents other than parents, such as teachers or peers. Prior research suggests that parental mediation is most impactful during early childhood, particularly up to 12 years of age [98,99], whereas our sample includes adolescents aged 11-17 years, a stage when peer influence tends to surpass that of parents [100]. In the context of active mediation, Shin and Lwin [101] found that peer and teacher guidance, rather than parental mediation, was linked to adolescents’ experiences with online risks. Similarly, in the health domain, advice from peers, but not from family members, was positively associated with physical activity among adolescents with overweight [102]. These findings suggest that peer eHealth literacy mediation may play an increasingly important role during adolescence and should be considered in future research. Additionally, the timing of our data collection during the COVID-19 pandemic, a period marked by heightened health anxiety and widespread health information exposure [103,104], may have amplified adolescents’ access to diverse sources of health guidance, potentially diminishing the relative influence of parental mediation.

Our study did not find support for the reverse effect, meaning that changes in adolescents’ eHealth literacy were not followed by corresponding changes in parental eHealth literacy mediation. One possible explanation is that parents may assume their children are already receiving adequate guidance from peers and teachers and thus perceive no need for additional mediation [105]. In the eHealth domain, this may be further exacerbated by parents’ limited confidence or expertise [106], which might lead them to delegate responsibility to schools or other professionals. Additionally, our assumption that parents would adjust their mediation based on their child’s needs may not hold if parents struggle to recognize those needs. Qualitative studies show that parents often lack insight into what their adolescent children do online [107], and adolescents frequently withhold information about their online challenges [108]. In the Czech Republic, more than half of adolescents rarely or never discuss their online activities with their parents [25], which can hinder parents’ ability to tailor mediation strategies effectively.

Additionally, our assumption relied on parents recognizing their children’s current level of eHealth literacy. We assumed that when parents perceive a need (eg, a low level of eHealth literacy), they would engage in mediation. However, accurately recognizing their children’s literacy level in this domain may not be easy. Qualitative studies show that parents often report lacking the knowledge or understanding of what their adolescent children do online [107]. In addition, adolescents often do not confide in their parents about their struggles and negative online experiences [108]; therefore, parents may not be fully aware of their child’s actual eHealth literacy levels or the specific challenges they face online, which could limit their ability to adjust mediation strategies effectively. In the Czech Republic, more than half of adolescents rarely or never talk to their parents about their online activities [25]. As a result, parents may easily miss important cues that would signal the need to enhance their eHealth literacy.

In our study, we examined the moderating effect of parental education on the relationship between eHealth literacy mediation and adolescents’ eHealth literacy. Previous research has shown that parents with higher education levels tend to be more confident in their digital skills and can engage more effectively in active mediation on various digital topics [21,50]. However, our findings did not support the moderating effect of parental education. There was no significant relationship between eHealth literacy mediation and adolescents’ eHealth literacy across any of the education groups. This suggests that higher parental education does not enhance the effectiveness of eHealth literacy mediation for this adolescent age group. One possible explanation is that although higher education is generally associated with more effective mediation, better-educated parents may overestimate their children’s digital competence, leading to less frequent or less engaged mediation. For example, Livingstone et al [21] observed that highly educated parents tend to assume their children are more digitally skilled than they actually are. Similarly, Kuldas et al [109] noted that parents with higher self-efficacy—often linked to higher education—may underestimate the frequency of their children’s online risk experiences, which can undermine the accuracy and responsiveness of their mediation strategies.

Future research could explore other potential moderators on the parental side, such as the quality of the parent-adolescent relationship. It is possible that the effectiveness of parental mediation is more closely linked to positive family relationships than to parents’ education or knowledge levels. Future research should therefore examine the complex interplay between parental awareness, adolescent disclosure, and family dynamics, as well as explore alternative socialization agents—such as peers, schools, or digital tools—that might effectively support adolescents’ eHealth literacy development.

### Methodological Considerations

Previous studies have primarily relied on cross-sectional research, demonstrating associations between constructs and differences among adolescents (ie, the between-person approach). Our study is innovative in that it examines individual-level changes within a longitudinal framework, presenting both within- and between-person results. Similar to previous research on adolescents’ digital skills [50], our study observed a between-person association, indicating that adolescents with higher eHealth literacy experienced more frequent eHealth literacy mediation. This finding is important because it highlights the distinction between the aforesaid 2 approaches in our study. Our study shows that in families with higher levels of eHealth literacy mediation, adolescents also have higher eHealth literacy. These 2 factors tend to co-occur. However, changes in eHealth literacy mediation do not correspond to changes in adolescents’ eHealth literacy within our sample. This finding has 2 important implications. First, this highlights the need for researchers and policy makers to exercise caution when relying solely on cross-sectional findings. Studies on parental mediation (and media effects more broadly) should increasingly use within-person methods if the field aims to generate more robust evidence to advance theory and inform evidence-based policy. Second, this finding suggests that—at least for early and middle adolescents—factors other than parental mediation play a more significant role in shaping their level of eHealth literacy.

### Limitations and Future Directions

One limitation of our study is the reliance on self-reported data from both adolescents and their parents. A potential shortcoming is that, due to social desirability bias, respondents might provide answers they believe are expected of them. Additionally, the tendency to overestimate or underestimate one’s own eHealth literacy could affect the accuracy of our findings. Similarly, the reported frequency of discussions about online health information may not fully capture the quality or effectiveness of those conversations. Future research could address these limitations by incorporating performance-based assessments, such as practical tasks or quizzes, to provide a more objective measure of eHealth literacy and minimize the biases inherent in self-reported data. Furthermore, although quota sampling was used to ensure demographic representativeness, the reliance on an online panel may have attracted participants with above-average digital engagement and skills, potentially underrepresenting individuals with limited internet access or lower digital literacy.

Another potential limitation is the 6-month interval between data collection waves for eHealth literacy mediation and adolescents’ eHealth literacy, which may have been too long. A shorter interval might have been more effective in capturing the potential impact of parental mediation. A follow-up study could also explore other factors that contribute to adolescents’ eHealth literacy. For instance, examining parental attitudes and behaviors toward digital technologies—alongside their own eHealth literacy levels and digital skills—may offer deeper insights into how eHealth literacy is transmitted from parents to children. In our study, we assessed parental education using a simplified classification (no diploma, diploma, and university). However, we acknowledge that this approach may not fully capture important aspects such as the parents’ field of study or alternative forms of education. Future research could improve upon this by more clearly distinguishing between different types of educational experiences, which may contribute differently to digital and eHealth literacy. Moreover, as educational background does not always equate to digital competence, examining digital skills as a distinct factor could offer a more nuanced understanding of the mediation process. Additionally, investigating broader familial dynamics, such as the quality of the parent-adolescent relationship, communication patterns, and parental role modeling of eHealth behaviors, could provide valuable insights into how the family context shapes adolescents’ engagement with online health information and the development of their eHealth literacy.

Future research could also benefit from distinguishing between different types of online health information that adolescents engage with, such as fitness, mental health, sexual health, or nutrition. These domains may not only attract varying levels of adolescent eHealth literacy and trust but may also elicit different parental mediation strategies. Disaggregating eHealth literacy by topic may, therefore, offer a more detailed understanding of how adolescents navigate specific eHealth domains and how parents support or influence these processes. Regarding age, future studies could also explore whether the relationship between parental mediation and adolescent eHealth literacy follows a nonlinear pattern. Testing for curvilinear associations could provide more nuanced insights into how mediation functions across developmental stages.

Lastly, we address the issue of sample attrition and the associated potential loss of statistical power. This may have reduced the likelihood of detecting small within-person effects (β=.10), resulting in less certain estimates. However, most of the observed effects were weaker than this threshold, indicating negligible effect sizes. Further, RI-CLPM has the advantage of decomposing variance into between- and within-person components [93], which reduces the likelihood of detecting spurious effects and generally provides less biased estimates. As most of the existing literature is based on cross-sectional data, which inherently cannot distinguish between these sources of variance, we consider our results to be an important contribution. We also acknowledge the use of manifest variables instead of multiple-indicator latent variables in our model. This choice limits the precision of our estimates, as measurement error was not controlled. However, because these variables did not represent multidimensional constructs and were primarily used to establish directional relationships, we still consider our results a valuable contribution. Nonetheless, we recommend that future research apply RI-CLPM with multiple indicators whenever possible to enhance measurement accuracy.

### Conclusions

This study explored the reciprocal dynamics between eHealth literacy mediation and adolescents’ eHealth literacy using a longitudinal research design. While no within-person reciprocal associations were found, noteworthy between-person associations emerged. This means that adolescents in families with higher eHealth mediation tend to have higher eHealth literacy; however, changes in eHealth literacy mediation do not appear to affect changes in adolescents’ eHealth literacy. Contrary to expectations, the study did not confirm a moderating role of parental education level. Our findings suggest that other factors may influence adolescents’ eHealth literacy, highlighting the need for further research. These results challenge the widely held theoretical assumption—based largely on cross-sectional studies—that parental mediation causally affects adolescents’ digital literacy and skills. The study’s results suggest that such associations may overestimate the true impact of parental mediation over time and underscore the need for more nuanced models that clarify when, how, and for whom parental mediation is effective.

## Appendix

Multimedia Appendix 1 Open Science Framework data.

Multimedia Appendix 2 Open Science Framework script.

## Abbreviations

ICT: information and communication technology
NUTS: Nomenclature of Territorial Units for Statistics
RI-CLPM: random intercept cross-lagged panel model

## Data Availability

The data used in this study originate from the Czech Science Foundation project no. 19-27828X. All data generated or analyzed during the study are included in this published article and its multimedia appendices.
